# Germline deletion of chromosome 2p16-21 associated with Lynch syndrome

**DOI:** 10.1038/s41439-021-00152-y

**Published:** 2021-05-19

**Authors:** Soichiro Natsume, Tatsuro Yamaguchi, Hidetaka Eguchi, Yasushi Okazaki, Shin-ichiro Horiguchi, Hideyuki Ishida

**Affiliations:** 1 Department of Surgery, Tokyo Metropolitan Cancer and Infectious Diseases Center Komagome Hospital, Tokyo, Japan; 2 Department of Clinical Genetics, Tokyo Metropolitan Cancer and Infectious Diseases Center Komagome Hospital, Tokyo, Japan; 3 Diagnosis and Therapeutics of Intractable Diseases and Intractable Disease Research Center, Juntendo University Graduate School of Medicine, Tokyo, Japan; 4 Department of Pathology, Tokyo Metropolitan Cancer and Infectious Diseases Center Komagome Hospital, Tokyo, Japan; 5 Department of Digestive Tract and General Surgery, Saitama Medical Center, Saitama Medical University, Kawagoe, Japan

**Keywords:** Cancer genetics, Colon cancer

## Abstract

We identified a Japanese patient with Lynch syndrome with a novel large germline deletion of chromosome 2p16-21, including the *EPCAM, MSH2*, and *KCNK12* genes. The proband was a 46-year-old man with ascending colon cancer. The clinical significance of germline *KCNK12* gene deletion, which encodes one of the subfamilies of two-pore-domain potassium channels, is still unknown.

Lynch syndrome (LS) is an autosomal dominant inherited disorder caused by a germline pathogenic variant in mismatch repair (MMR) genes, including the *MLH1*, *MSH2*, *MSH6*, and *PMS2* genes. Inactivation of the MMR gene, which is caused by both germline and/or somatic variants, leads to an increased frequency of replication errors in repeated sequences in the coding regions of cancer-related genes, resulting in the development of LS-associated tumors^1^. Therefore, LS is characterized by the development of various cancers at a young age, including colorectal cancer, endometrial cancer, small bowel cancer, and urinary tract cancer.

The *EPCAM* gene, which is located 17 kb upstream of *MSH2*, is not an MMR gene. However, germline deletion of the 3’ end of the *EPCAM* gene causes LS because *EPCAM* deletion leads to *MSH2* gene silencing by promoter hypermethylation and *MSH2* inactivation^2^. The proportion of *EPCAM* gene deletions in LS is estimated to be 1.0–3.0%^3,4^, and to date, only a few families have been reported in Japan^5^.

Here, we identified a Japanese patient with LS with a novel large germline deletion of chromosome 2p16-21, including the *EPCAM, MSH2, KCNK12* genes. This novel finding provides evidence of germline deletion in this region and raises questions regarding the deletion of the *KCNK12* gene.

The proband was a 46-year-old man who was referred to our hospital with abdominal pain. A colonoscopy demonstrated an ascending colonic tumor, and biopsy revealed adenocarcinoma in the ascending colon. However, computed tomography showed no evidence of lymph node metastasis, distant metastasis, or other organ diseases. In addition, his mother had been treated for colon cancer, glioblastoma, gastric cancer, and endometrial cancer, and she died from glioblastoma at the age of 67 years old (Fig. 1).

**Fig. 1:**
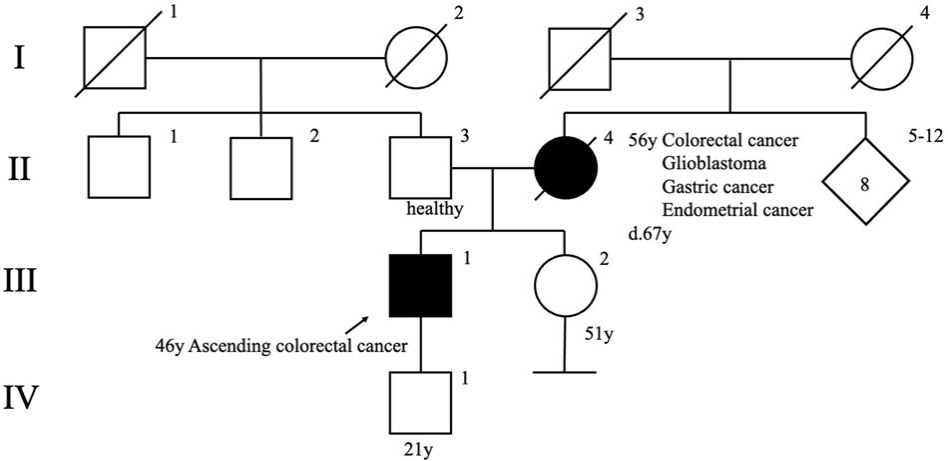
Pedigree of the patients. The arrow indicates the proband. A filled symbol indicates a person with colorectal cancer.

The patient underwent laparoscopic right hemicolectomy, and histopathological examination revealed moderately differentiated adenocarcinoma with a mucinous component in the ascending colon tumor.

After obtaining the patient’s informed consent, we analyzed the patient’s tumor and found a high frequency of microsatellite instability. Then, after genetic counseling, we performed genetic analysis using next-generation sequencing^6^. The assay detected deletion of the *EPCAM* and *MSH2* genes. We conducted further analysis using multiplex ligation-dependent probe amplification (MLPA) to confirm the deletion. Assays were performed to detect large genomic rearrangements of the *EPCAM* and *MSH2* genes using an *MLH1/MSH2* MLPA kit following the manufacturer’s protocol (SALSA MLPA KIT P-003 MLH1/MSH2, MRC-Holland, Amsterdam, The Netherlands). Then, we detected germline deletions of all *MSH2* exons and exon 9 of the *EPCAM* gene. An additional assay was performed using an *MSH6* MLPA kit (SALSA MLPA KIT P-072 MSH6), and we detected the deletion of the *EPCAM* gene and exon 2 of the *KCNK12* gene, which was located upstream of the *EPCAM* gene (Fig. 2a, b).

**Fig. 2:**
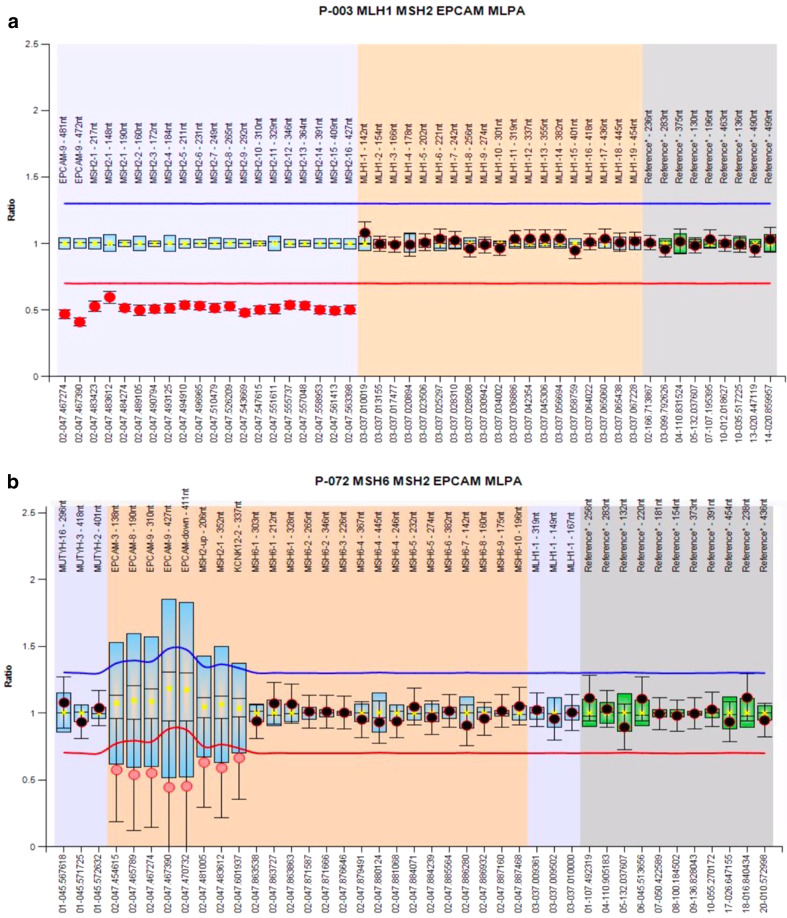
Multiplex ligation-dependent probe amplification (MLPA) analysis of mismatch repair genes. Ratio chart for MLPA analysis using SALSA MLPA Probe Mix P-003 (**a**) and P-072 (**b**) demonstrating germline deletions of the *EPCAM*, *MSH2*, and *KCNK12* genes.

We identified a novel germline deletion of chromosome 2p16-21, including the *EPCAM*, *MSH2*, and *KCNK12* genes. Large genomic deletions and duplications, such as this one, have recently been identified using MLPA^5^. However, to our knowledge, there are no reports of germline deletions in this region.

The *MSH2* gene is more likely to be found in genomic rearrangements at the locus of this disease because the *MSH2* gene has a higher number and density of Alu repeats than other MMR genes^7^. At least 20% of germline *MSH2* pathogenic variants are deletions of exons or multiple exons.

*MSH2* variant carriers have a significantly higher risk of developing cancer of the urinary tract compared with carriers of *MLH1* and *MSH6* mutations^8^. Moreover, male *MSH2* variant carriers have a high risk of developing cancer of the stomach^9^. However, our patient and his relatives had not developed urinary tract cancer or stomach cancer.

The difference in cancer risks between *EPCAM* deletion carriers and *MSH2* variant carriers has been reported. The cumulative risk of endometrial cancer in *EPCAM* deletion carriers is lower than that in *MSH2* variant carriers, while the cumulative risk of colorectal cancer in *EPCAM* deletion carriers is similar to that in *MSH2* variant carriers^10^. On the other hand, the cumulative risk of colorectal cancer and endometrial cancer in carriers of a concurrent deletion of the *EPCAM* and *MSH2* genes is reported to be similar to that in *MSH2* variant carriers^10^. However, none of our patient’s relatives had developed endometrial cancer. In our study, to confirm the role of MMR proteins in carcinogenesis, immunohistochemical staining for MSH2 and EPCAM was conducted on colorectal cancer tissue, which showed only the loss of MSH2 expression (Fig. 3a, b). Thus, in our patient’s colorectal cancer, dysfunction of the MSH2 protein caused dysfunction of the MMR system, followed by a DNA replication error. Therefore, if the cancer risks were different between the *MSH2* and *EPCAM* genes, the expression of MMR proteins should also be considered.

**Fig. 3:**
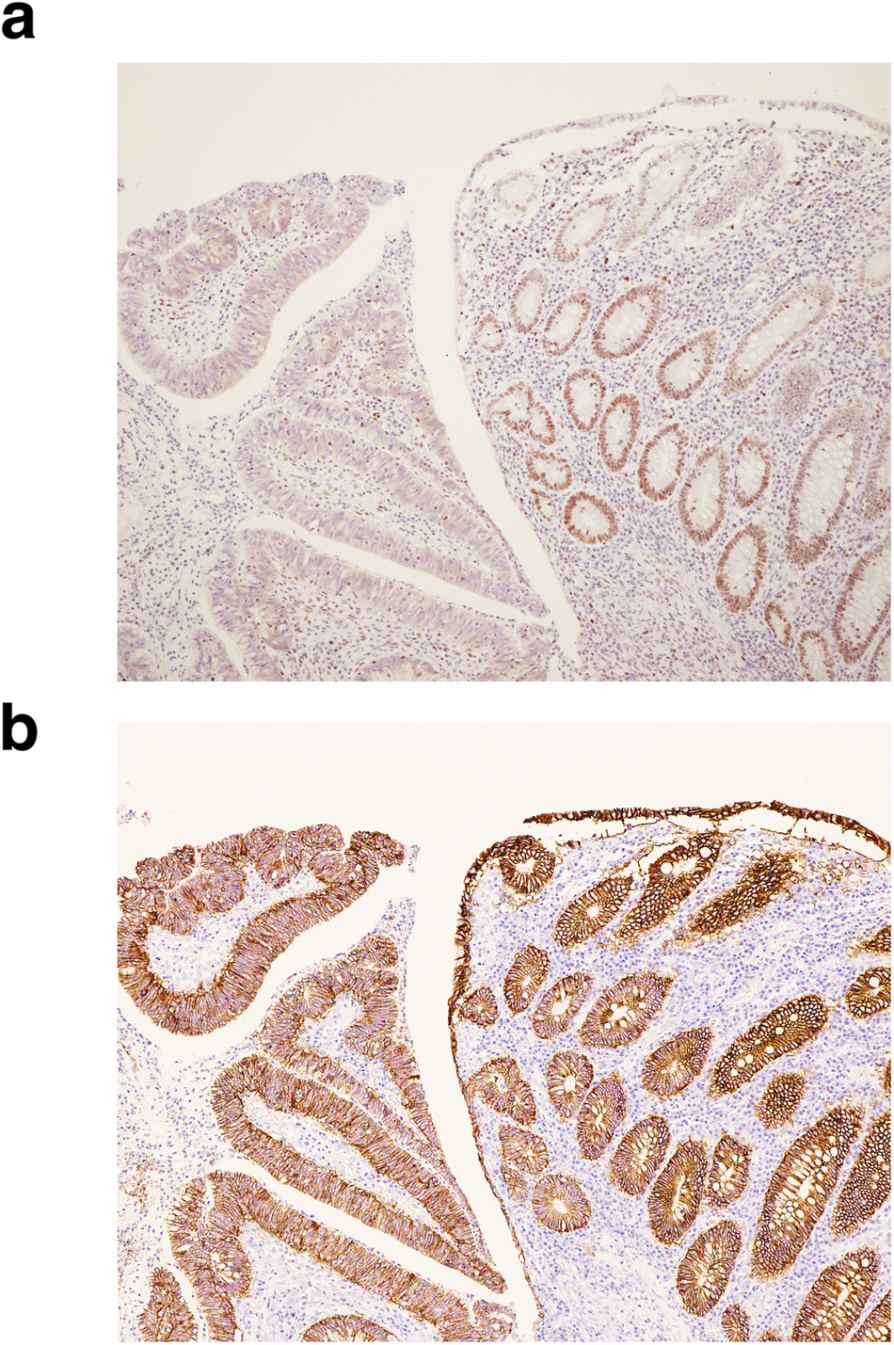
Immunohistochemistry for mismatch repair protein in ascending colon cancer. Tumor tissue showed loss of staining for MSH2 (**a**) while staining is retained for EPCAM (**b**).

The *KCNK12* gene located on 2p22-2p21 encodes one of the subfamilies of two-pore-domain potassium channels^11^. *KCNK12* expression has been found in various organs, such as the pancreas, heart, skeletal muscle, ovary, testis, prostate, colon, peripheral blood leukocytes, small intestine, spleen, and thymus^11^. By setting and modulating the cellular membrane potential, potassium channels play a significant role in neuronal activity, muscular excitability, and hormone secretion^12^. Recent evidence supports the hypothesis that alterations in the expression and function of two-pore-domain potassium channels may contribute to cancer development and progression^13^, including breast cancer, colorectal cancer, leukemia, and lymphoma^14–16^. Further, a single-nucleotide polymorphism within the *KCNK12* gene has been identified as a candidate genetic marker for migraine in the Finnish population^17^. However, our patient and his relatives did not have migraines or a neurovascular disorder.

The limitations of this report include the absence of segregation analysis and no identification of a breakpoint or rearranged genome. In conclusion, we identified a novel large germline deletion of chromosome 2p16-21, including the *EPCAM*, *MSH2*, and *KCNK12* genes. However, the clinical significance of germline deletion of the *KCNK12* gene is still unknown. Therefore, it is necessary to obtain and examine more data on this topic.
